# Optimizing Recovery: A Comprehensive Case Report on Physiotherapy Rehabilitation in Esophagectomy Patients

**DOI:** 10.7759/cureus.63473

**Published:** 2024-06-29

**Authors:** Anandi R Dave, Tejaswini B Fating

**Affiliations:** 1 Community Health Physiotherapy, Ravi Nair Physiotherapy College, Datta Meghe Institute of Higher Education and Research, Wardha, IND

**Keywords:** transthoracic esophagectomy, community physiotherapy, post-operative mobility, respiratory physiotherapy, oncology rehabilitation, physical therapy rehabilitation, carcinoma esophagus

## Abstract

Esophageal carcinoma (CA) represents a significant global health risk, attributable to its origin from esophageal epithelium, among many other associated risk factors. Its alarming rise in younger age groups, especially among females, is concerning, even though historically, it has been more common in older populations. This modification emphasizes how complex the interaction of genetic susceptibility, environmental factors, and lifestyle choices is in determining the course of a disease. It is impossible to overstate the importance of an early diagnosis and multidisciplinary care, especially for younger patients where delayed detection is expected. Through the use of evidence-based practices, physical therapy has emerged as a crucial part of the overall care of patients with esophageal cancer. The six-minute walk test (6MWT), a popular physiotherapy evaluation tool, can be used to evaluate functional ability and exercise tolerance. Understanding how well younger people can exercise using the 6MWT is significant since they have more excellent exercise capacity than older people. This test helps physiotherapists evaluate the improvement of a patient's exercise capacity before and after the rehabilitation. In this case study, the 31-year-old woman's incredible recovery from esophageal cancer was made possible by extensive cardio-respiratory physiotherapy rehabilitation, demonstrating the significant influence of this physiotherapeutic intervention on functional status and general well-being. Through this study, we contribute to the advancement of scientific knowledge as well as the caring, patient-centered ideology that guides oncology treatment today.

## Introduction

The lethal disease known as esophageal carcinoma (CA), which arises from the esophageal epithelium, has a significant influence on global health [1]. Although it has previously been seen in older people, its emergence in younger demographics, especially women, has become a concerning pattern [2]. The intricate interplay of lifestyle choices, environmental factors, and genetic predisposition underscores the intricacy of the condition. When it comes to treating esophageal cancer, prompt diagnosis and comprehensive multidisciplinary care are essential, particularly in younger patients, as the disease's rarity usually causes delayed identification [3,4]. Patients with esophageal cancer receive extensive care in this setting that goes much beyond standard medical procedures. It covers a wider variety of therapeutic approaches, with physiotherapy acting as a foundational treatment. Physiotherapy-based therapies grounded on evidence-based practice are employed to address the different challenges patients face [5,6].

From addressing physical ailments to enhancing the general quality of life (QoL), physiotherapy might assist [7,8]. Of all the physiotherapy assessments, the six-minute walk test (6MWT) is one of the most effective. This simple yet informative examination evaluates a patient's functional ability and exercise tolerance [9,10]. In the case of esophageal cancer, when fatigue, dyspnea, and physical deconditioning are prevalent, the 6MWT is quite helpful. It evaluates the patient's reaction to physical activity, cardiovascular endurance, and fitness [11-13].

Because young individuals with esophageal cancer frequently have greater baseline functional capacities, the 6MWT is particularly relevant in the comprehensive care of this patient population. It is vital to track how these patients respond to therapy and physical rehabilitation [14,15]. By using the 6MWT better to understand the subtle aspects of their exercise tolerance, physiotherapists can create safe and effective treatments that lead to optimal results and enhanced QoL [16]. An essential factor in the 31-year-old girl's recovery from esophageal cancer was the 6MWT. By exploring its nuances, we want to shed light on the potentially transformative impact of this physiotherapeutic intervention on the functional status and overall well-being of young individuals coping with this challenging illness. Our research adds to the body of scientific knowledge while advancing the patient-centered, compassionate approach that is the basis for modern oncological care.

## Case presentation

Patient information

A female patient, aged 31, came to the tertiary care hospital complaining of dysphagia that had been persistent for two years. There was no history of vomiting or nausea. The patient had esophageal CA and had undergone four rounds of chemotherapy. When evaluating the location, size, and appearance of a tumor in an adult CA esophageal instance, endoscopy was recommended. The histological analysis of biopsy samples was recorded. A 26 cm-long ulceroproliferative friable tumor was discovered during an endoscopy, and an imaging test such as contrast-enhanced computed tomography (CECT) thorax showed a calcified lymph node measuring 1.3 cm x 0.8 cm. Later, she went through a planned esophagectomy due to a failed response to chemotherapy. The esophagectomy was transthoracic. An anastomotic leak or infection did not appear to be present. In laboratory examinations, a considerable decrease in the total count of white blood cells was seen, together with macrocytic anemia (hemoglobin was 9 g/dL) manifesting as pallor with a blood pressure recording of 130/90 mmHg and a heart rate of 94 beats per minute noticed during a routine assessment. After surgery, she had respiratory distress, limited mobility, and pain that required extensive physiotherapy.

Clinical findings

Informed consent was obtained before assessment and treatment. The patient was well-oriented to time, place, and person. Chest expansion was 1 cm on both axillary and xiphoid levels, which is reduced. Breathing too quickly and shallowly and having weaker cough reflexes after surgery make it harder to get rid of mucus and secretions from the airways, which raises the risk of respiratory infections. Auscultation for breath sounds was done, which showed reduced air entry and fine crackles in bilateral lower zones.

Diagnostic assessment

**Figure 1:**
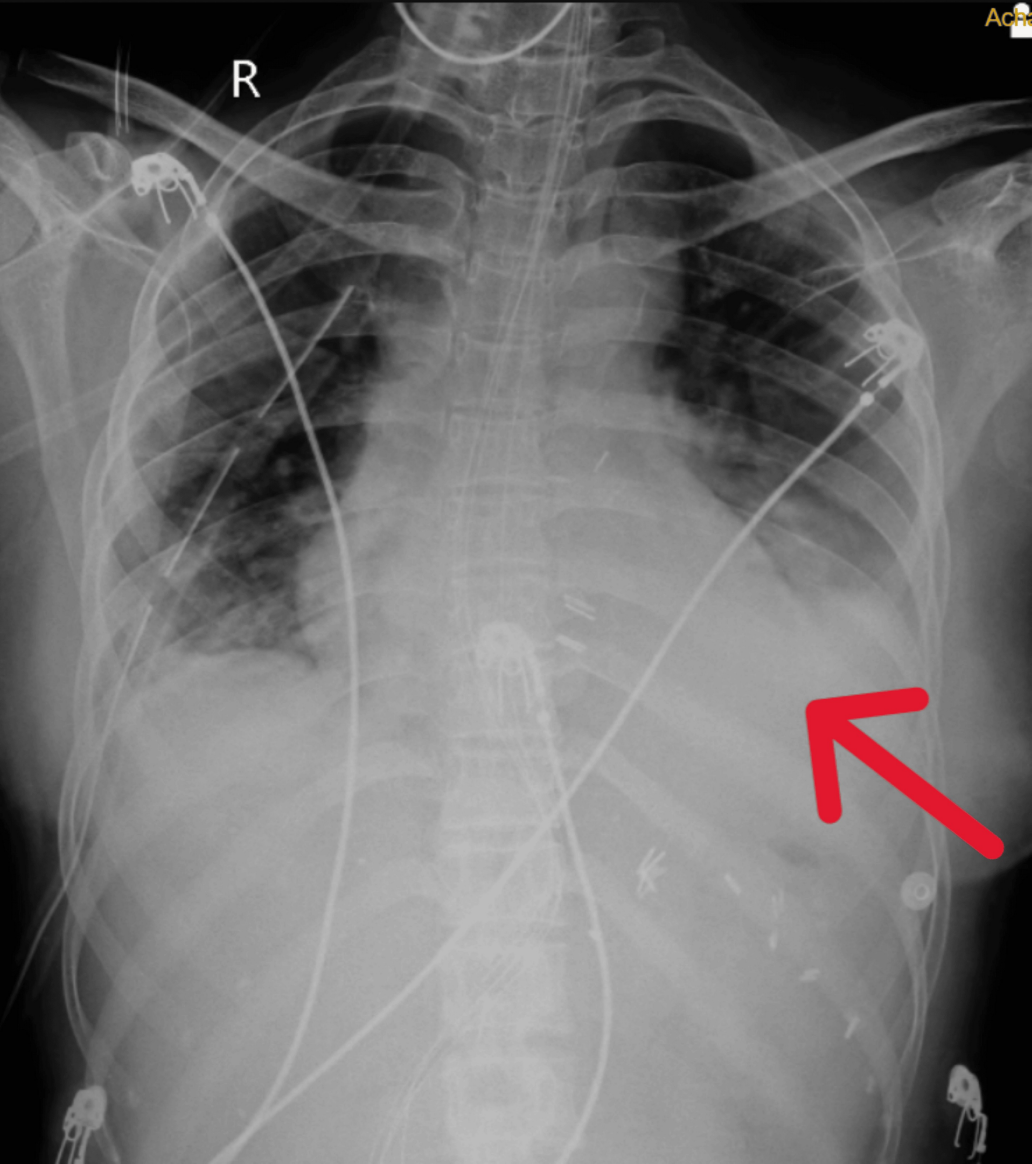
X-ray PA view of pre-rehabilitation The red arrow shows homogenous opacity. PA: posteroanterior view

**Figure 2:**
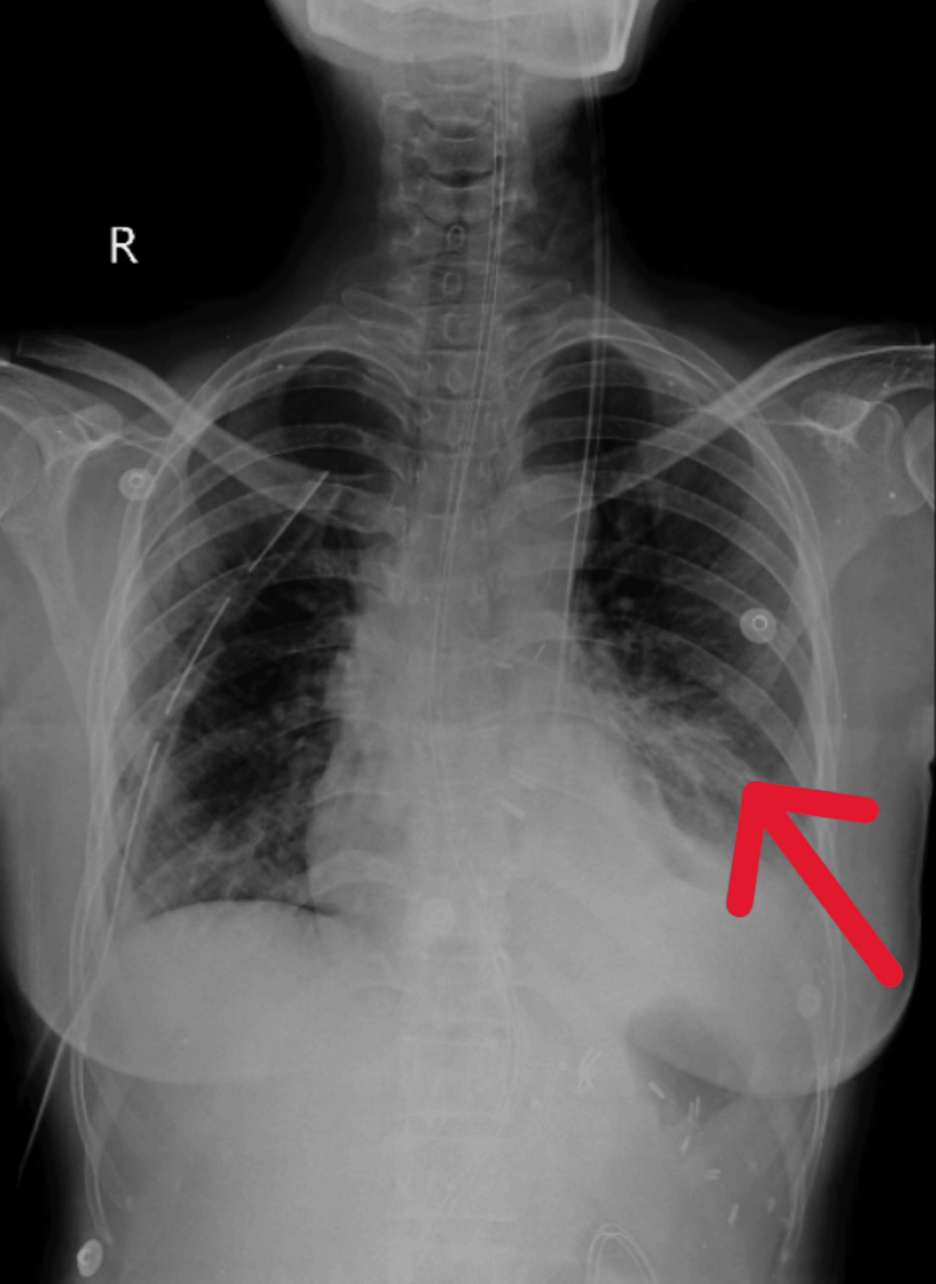
X-ray AP view of post-rehabilitation The red arrow shows the improved lung field. AP: anteroposterior view

The patient's chest X-ray was done before starting the physiotherapy intervention, as shown in Figure 1. After four weeks of extensive physiotherapy rehabilitation chest X-ray was obtained on post-operative day 30, as shown in Figure 2.

Physiotherapy intervention

An extensive physiotherapy rehabilitation was started immediately following the surgery. The rehabilitation was extended to four weeks. The physiotherapy program was tailored to the patient's needs, emphasizing breathing exercises, chest physiotherapy, early mobilization, and pain management. The protocol is mentioned in Table 1. gfv

**Table 1:** Rehabilitation protocol cc: cubic centimeter; TENS: transcutaneous electrical nerve stimulation; QoL: quality of life

Goals	Interventions	Dosage/frequency
Improve respiratory function	Deep breathing exercises	5-10 minutes, 3 times/day
	Diaphragmatic breathing (Increase the overall lung volume and enhance the ability to take deeper breaths)	10 minutes, 2-3 times/day
	Incentive spirometry	600 cc with 1 second hold and progressed to 1200 cc with 2 seconds hold
	Airway clearance techniques (if necessary)	1-2 times/day
Enhance swallowing function	Swallowing exercises (supervised by therapist)	2-3 times/day
	Postural training	Integrated into daily activities
	Compensatory strategies (head rotation, chin tuck, head tilt, bolus viscosity, texture, and volume modifications) [17]	During daily mealtime
	Supraglottic swallow, super-supraglottic swallow	During daily mealtime
Pain management	Gentle mobilization and stretching of affected muscles	10-15 minutes, 2-3 times/day
	Modalities (heat/cold therapy and TENS) as needed	12 minutes
Improve QoL	Fatigue management	Energy conservation techniques
	Relaxation techniques (deep breathing)	5-10 minutes, 2 times/day
	Emotional support and counseling	Provided by a counselor

**Figure 3:**
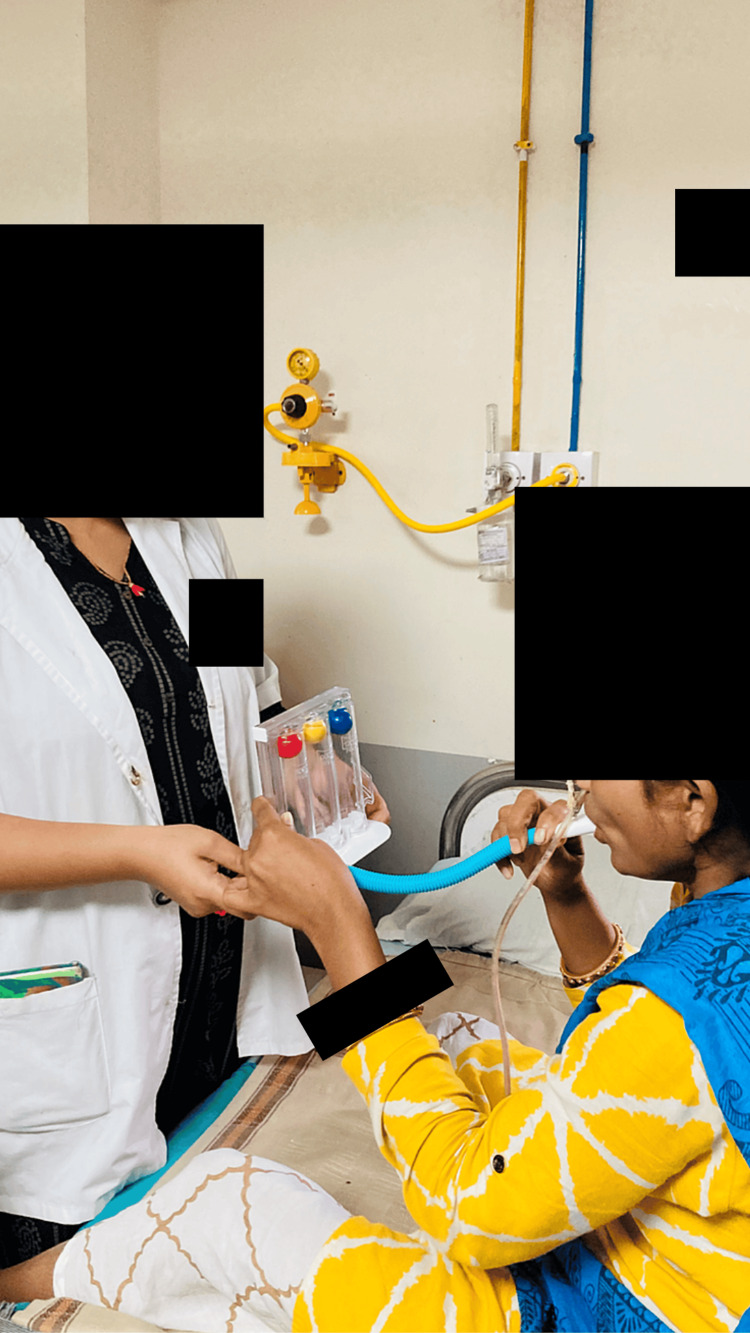
Incentive spirometry A three-chamber spirometer is a simple and effective device used to encourage deep breathing, often post-operatively, to prevent lung complications such as atelectasis or pneumonia. It involves deep inhalation to lift the balls or pistons, holding the breath, and exhaling slowly. The results are interpreted based on the volume of air inhaled, which helps gauge lung function and recovery. Regular use of the spirometer is crucial for improving lung capacity and preventing complications, especially after surgery. Typical volume ranges: below 1500 mL: generally considered low and may require focused respiratory therapy; between 1500 and 2500 mL: moderate, often expected in the initial post-operative period or in individuals with mild to moderate lung impairment; and above 2500 mL: indicates good lung function, often a target for healthy individuals.

**Figure 4:**
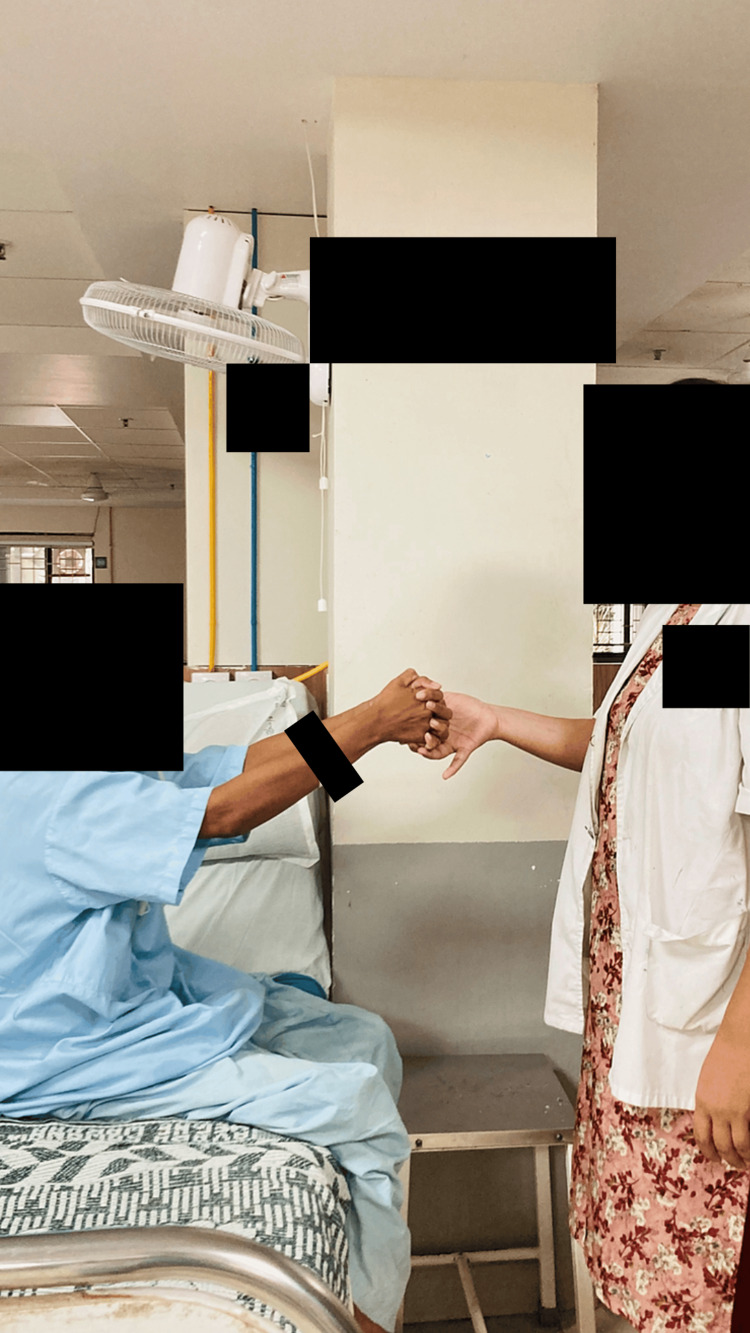
Thoracic expansion exercise

Figure 3 and Figure 4 show some of the rehabilitation exercises such as incentive spirometry and thoracic expansion, respectively. 

The right ICD was removed on post-operative day 12. No signs of discomfort were seen hence the rehabilitation was resumed after one day.

Outcome measure

The patient's ability to participate in functional activity is assessed using the 6MWT. The patient's total cardiovascular endurance and fitness are measured using their 6MWT distance. Measurements were taken both before and after surgery to determine how much the patient's condition had improved. Prior to rehabilitation, the score was 88.74%; following rehabilitation, the score was 92.2%.

## Discussion

The case study of a 31-year-old female patient who underwent esophagectomy and subsequent physiotherapy rehabilitation provides essential insights into the multifaceted approach required for optimal recovery following such a significant surgical intervention. This discussion highlights the critical role of physiotherapy in these cases, the challenges encountered, and the broader implications for patient care.

Physiotherapy is indispensable in the post-operative care of esophagectomy patients. It encompasses a range of interventions, including pre-operative education, breathing exercises, coughing techniques, early mobilization, and ongoing post-operative follow-up. These interventions are designed to prevent pulmonary complications, enhance physical function, and improve overall recovery outcomes.

One of the primary challenges in providing effective physiotherapy care for esophagectomy patients is the lack of standardized protocols and evidence-based practices. Hussey et al. (2018) investigated the current physiotherapy regimen for Swedish patients undergoing esophageal cancer surgery. They found that while most physiotherapists offered essential interventions such as pre-operative education, breathing exercises, and early mobilization, there was a notable deficiency in standardized outcome measures and evaluation techniques. This gap highlights the need for further research and collaboration to enhance the quality and effectiveness of physiotherapy care in this patient population [18].

The benefits of physiotherapy interventions in esophagectomy and other upper gastrointestinal surgeries are well-documented. Tukanova K et al. (2021) conducted a meta-analysis of 24 studies, including 12 randomized controlled trials and 12 cohort studies, to evaluate the advantages of physical therapy regimens for patients undergoing gastrectomy or esophagectomy. The findings demonstrated that rehabilitation exercise interventions conducted before surgery significantly reduced post-operative pneumonia rates and overall morbidity. Similarly, perioperative and post-operative rehabilitation exercise interventions after surgery were shown to lower pneumonia rates, reduce hospital stays, and improve QoL scores related to dyspnea and physical functioning [19].

The insights gained from this case study and supporting literature underscore the critical need for a comprehensive, evidence-based approach to physiotherapy in esophagectomy patients [20]. Prehabilitation can enhance patients' physical readiness for surgery, potentially leading to better post-operative outcomes. Early mobilization and breathing exercises are crucial in the immediate post-operative period to prevent complications such as atelectasis and pneumonia. Ongoing rehabilitation helps in regaining physical function and improving QoL.

However, the studies also indicate that more research is needed to fully understand the mechanisms behind these benefits and to identify the patient subgroups that would derive the most benefit from specific interventions. Tailoring physiotherapy regimens to individual patient needs, based on a thorough assessment and standardized evaluation techniques, is essential for optimizing recovery.

## Conclusions

The successful recovery from esophagectomy in this 31-year-old female patient underscores the critical role of physiotherapy in optimizing post-operative outcomes. Tailored interventions, including breathing exercises, chest physiotherapy, early mobilization, pain management, and emotional support, were pivotal in facilitating recovery and enhancing overall well-being. Early mobilization, despite challenges like hemodynamic instability associated with thoracic epidurals, proved essential for improving respiratory function, minimizing pain, and promoting quicker recovery. Goal-directed mobilization requiring close multidisciplinary collaboration, effectively addressed these challenges, ensuring safe and effective patient movement. This case study emphasizes the importance of customized rehabilitation programs tailored to the unique needs of esophagectomy patients. Continuous monitoring and early intervention, combined with addressing the psychological well-being of the patient, provided a holistic approach to recovery. The significant impact of multidisciplinary collaboration in overcoming post-operative mobilization challenges further highlights the value of coordinated care. Ultimately, the study demonstrates the profound impact of physiotherapy in aiding full and efficient recovery, reducing complications, and enhancing the QoL, serving as a model for optimizing post-operative care in esophagectomy patients.
